# Evaluation of natural smile: Golden proportion, RED or Golden percentage

**DOI:** 10.4103/0972-0707.43413

**Published:** 2008

**Authors:** B. V. Sreenivasan Murthy, Niketa Ramani

**Affiliations:** Department of Conservative Dentistry and Endodontics, MS Ramaiah Dental College, Bangalore, India

**Keywords:** Golden percentage, golden proportion, recurring esthetic dental

## Abstract

Creating geometric or mathematical proportion to relate the successive width of maxillary anterior teeth is a critical aspect in Esthetic dentistry. Golden proportion, recurring esthetic dental (RED) proportion and golden percentage are new theories in this field.

**Aim:**

To investigate the existence and suitability of Golden proportion, Recurring Esthetic Dental, and Golden percentage between the widths of maxillary anterior teeth in individuals with natural dentition, with the aid of digital photographs and computer analysis.

**Material and Methods:**

Standardized frontal images of 56 dental students, 20 male and 36 female, were captured. Each maxillary anterior tooth was digitally measured. Once the measurements were recorded, the three theories were applied and the data was analyzed statistically.

**Results:**

The golden proportion was found to exist only in 14-25% of the subjects, between perceived maxillary anterior teeth in natural dentition. The value of RED proportion was not constant, and as one moved distally, this proportion gradually increased.

Furthermore, the results revealed that golden percentage was rather constant in terms of relative tooth width. Central incisor represented 22%, lateral incisor 15% and canine 13% of the width of six maxillary anterior teeth, as viewed from the front.

**Conclusion:**

Both golden proportion and RED proportion are unsuitable methods to relate the successive width of the maxillary anterior teeth in natural dentition. However, the golden percentage theory can be applied if percentages are adjusted, taking into consideration the ethnicity of the population.

## INTRODUCTION

‘No human inquiry can be called science unless it pursues its path through mathematical exposition and demonstration.’ Leonardo da Vinci.

One of the critical aspects of esthetic dentistry is creating geometric or mathematical proportion to relate the successive width of anterior teeth. Golden proportion, golden percentage and recurring esthetic dental are theories introduced in this field.[1,2,4] Lombardi was the first to suggest the application of the golden proportion in dentistry. He said that the golden proportion was ‘too strong’ for use in determining tooth size.[1] He also described the use of a ‘repeated ratio’ in the maxillary anterior teeth. This implies that an optimized dentofacial composition of the lateral to central incisor width and the canine to lateral incisor width are repeated in proportion.[1] Levin suggested the use of the theory of Golden proportion to relate the successive width of the anterior teeth, as viewed from the labial aspect. He said that the width of the central incisor should be in golden proportion to the width of the lateral incisor and that the lateral incisor should be in golden proportion to the width of the canine, when viewed from the front.[2] In addition, he devised a grid with the spaces in golden proportion and advocated the use of this grid to evaluate and develop harmonious proportions of teeth.[3]

However, in a more recent study, it was reported that the golden proportion did not exist between the widths of the maxillary anterior teeth in individuals who have an esthetic smile.[3] Ward suggested the recurring esthetic dental (RED) proportion. He based his suggestion on the result of his study in which he described RED proportion as the proportion of the successive width of the teeth remaining constant, when progressing distally from the midline.[4] Snow considered a bilateral analysis of apparent individual tooth width as a percentage of the total apparent width of the six anterior teeth. He proposed the golden percentage, wherein the proportional width of each tooth should be: canine 10%, lateral 15%, central 25%, central 25%, lateral 15%, and canine 10% of the total distance across the anterior segment, in order to achieve an esthetically pleasing smile.[5]

### Aim

To investigate the existence and suitability of Golden proportion, Recurring Esthetic Dental, and Golden percentage between the widths of maxillary anterior teeth in individuals with natural dentition, with the aid of digital photographs and computer analysis.

## MATERIALS AND METHODS

### Subject selection

Fifty six dental students, 20 male students and 36 female students in the 20-25 age group, were selected for the study.

### Inclusion criteria

*Subjects* : Asian origin; natural dentition in maxillary anterior region.

*Exclusion criteria:* Subjects who have undergone orthodontic treatment; maxillary anterior tooth size alterations.

### Image capture

Standardized frontal image of each subject's smile was taken, using digital camera NIKON D100, AF MICRO NIKKORE, 105MM, in the following manner:

Subjects were positioned in the natural head position.

The camera was positioned and adjusted so as to obtain a sharp image of the face, from the tip of the nose to the tip of the chin. The distance between the camera and the subject was fixed at a working distance of 60 cm. The camera was stabilized with the help of a tripod, at this fixed distance.

The subject was asked to smile and the image was captured during the smile.

The images were then downloaded to a personal computer. All the measurements were taken with the help of the software Adobe Photoshop 7, by one investigator.

### Measurements

The Golden proportion for each subject was measured thus: the width of the central incisor was multiplied by 62% and compared with the width of adjacent lateral incisor. Similar values indicate that the width of the central incisor is in golden proportion to the width of the lateral incisor.

By comparing the width of the lateral incisor multiplied by 62% with that of the canine, it can be determined whether the width of the lateral incisor is in golden proportion to the width of the canine.

RED proportion was calculated by dividing the width of each lateral incisor by the width of the adjacent central incisor and the resulting number was multiplied by 100. Similarly, the width of each canine was divided by the width of adjacent lateral incisor and the resulting number was multiplied by 100. If the values obtained are constant, it means that the central incisor, lateral incisor, and canine are in RED proportion.

The golden percentage was calculated by dividing the width of each central incisor, lateral incisor and canine by the total width of all six maxillary anterior teeth and multiplying the resulting value by 100, in order to obtain the golden percentage for each tooth. If the values from canine to canine were 10, 15, 25, 25, 15, and 10%, it indicates that the six maxillary anterior teeth are in golden percentage.

The data was statistically analyzed using the paired T test *P* < .05 %.

## RESULTS

Table 1 gives the width of teeth starting from right canine to left canine.

**Table 1:** Table for width of teeth

SLN	SEX	CIWL	CIWR	LIWL	LIWR	CAWL	CAWR
1	M	9.4	9.65	6.77	6.94	5.42	5.42
2	M	11.5	11.6	7.54	7.7	6.01	5.73
3	M	9.1	8.47	6.7	6.6	5.59	5.25
4	M	8.8	9.31	7.2	6.35	5.08	4.91
5	M	10.8	10.75	6.35	7.2	5.59	5
6	M	9.8	10.08	7.7	7.7	6.52	5.84
7	M	11.2	10.08	9.4	8.13	7.37	6.77
8	M	9.2	9.57	5.16	6.07	5.08	6.01
9	M	8.6	8.74	6.54	5.59	5.08	6.01
10	M	10.3	9.99	7.54	6.77	6.69	6.94
11	M	9.8	9.99	5.76	6.01	5.59	5.54
12	M	10.2	10.41	6.77	7.96	5.08	6.77
13	M	10.4	10.33	5.84	6.77	5.59	6.77
14	M	10.3	9.9	6.77	7.37	5	5.42
15	M	10.3	10.58	6.77	7.2	6.35	6.69
16	M	10.2	9.82	7.79	7.37	6.01	6.43
17	M	11.8	11.37	8.13	7.03	5.84	5.25
18	M	9.8	9.99	7.02	6.94	6.01	5.84
19	M	10	10.58	7.7	7.79	5.25	5
20	M	9.7	9.82	7.11	7.77	5.42	6.01
21	F	9.5	9.57	6.35	7.28	5.08	5.25
22	F	10.6	10.33	6.69	5.76	5.84	4.83
23	F	11.4	12.02	8.3	7.54	6.43	5.67
24	F	10.2	9.48	6.18	6.94	5.16	6.27
25	F	9.4	9.9	7.28	7.11	4.23	5.84
26	F	8.3	8.64	6.01	6.18	5	5.84
27	F	9.5	9.48	6.43	5.67	5.93	6.43
28	F	8.1	8.21	5.25	4.74	4.83	4.4
29	F	11	11.77	7.62	7.2	5.42	6.35
30	F	8.7	8.97	6.69	6.94	5.42	5.42
31	F	10	9.57	5.67	6.69	5.42	6.86
32	F	9.4	9.14	6.18	6.77	5.42	5.93
33	F	9.6	9.65	7.7	6.6	6.6	6.6
34	F	9.1	8.89	6.94	7.11	5.25	5.42
35	F	9.6	9.4	6.01	6.18	4.83	4.23
36	F	9.4	10.41	6.6	6.18	5.08	4.66
37	F	9.6	10.08	7.11	7.11	5.25	5.42
38	F	10.2	9.41	7.2	6.43	5.25	4.4
39	F	9.4	9.31	6.6	6.94	4.66	5.42
40	F	9.8	10.16	6.6	7.7	4.91	5.67
41	F	9.4	9.82	6.18	7.28	4.32	5.67
42	F	9.2	8.89	6.77	6.77	5.08	5.42
43	F	10.2	9.65	6.43	6.35	6.01	5.76
44	F	12.2	9.31	5.59	6.27	5.42	5.08
45	F	9.1	9.06	6.77	7.37	5.42	5
46	F	9.4	9.06	6.01	5.84	5	4.66

CIWL= Central incisor width left side. CIWR= Central incisor width right side. LIWL= Lateral incisor width left side. LIWR=Lateral incisor width right side. CAWL= Canine width left side. CAWR=Canine width right side.

A cut off value was arrived at, to determine whether the subjects lie in the golden proportion range or not. The cut-off value was calculated as follows:

First, the difference between two groups was calculated, following which an average mean was calculated. Once the average mean was derived, values lying within the range of average mean + 1 Standard Error was considered to be in golden proportion.

**Graph 1:**
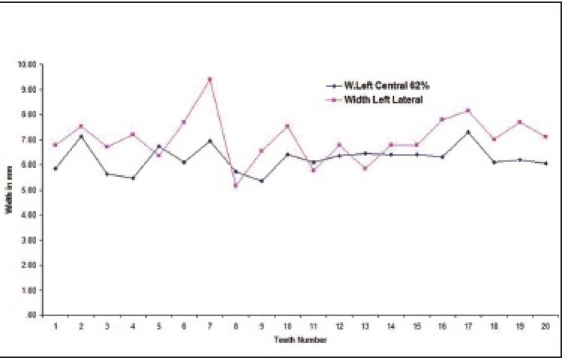
Indicates golden proportion relation between left central incisor (blue line) and left lateral incisor (red line) in male subjects

**Graph 2:**
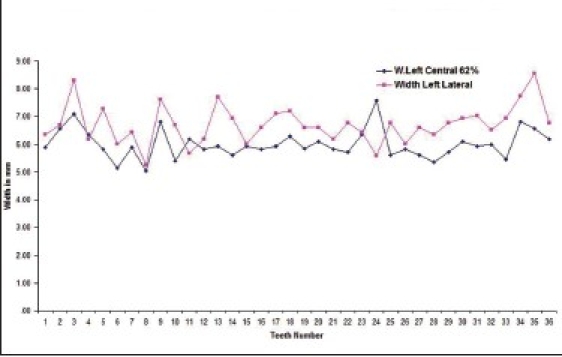
Indicates golden proportion relation between left central incisor (blue line) and left lateral incisor (red line) in female subjects

Out of the total subjects, 17.9% had left central incisor in golden proportion to left lateral incisor [Graphs 1 and 2 respectively].

**Graph 3:**
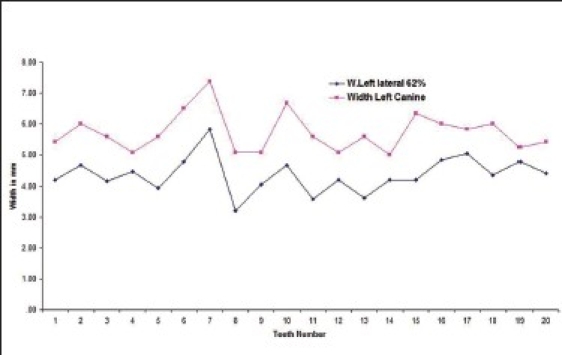
Golden proportion relation between left lateral incisor (blue line) and left canine (red line) in male subjects.

**Graph 4:**
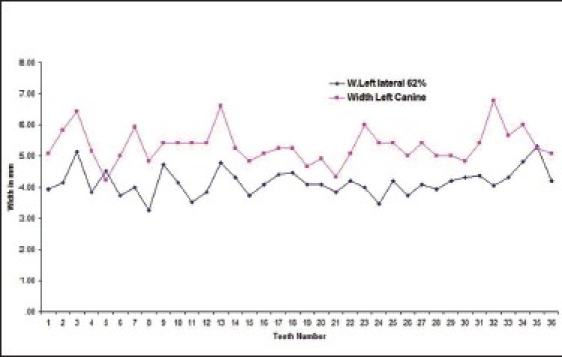
Golden proportion relation between left lateral incisor (blue line) and left canine (red line) in female subjects).

Twenty five percent of the subjects had left lateral incisor in golden proportion to left canines [Graphs 3 and 4 respectively].

**Graph 5:**
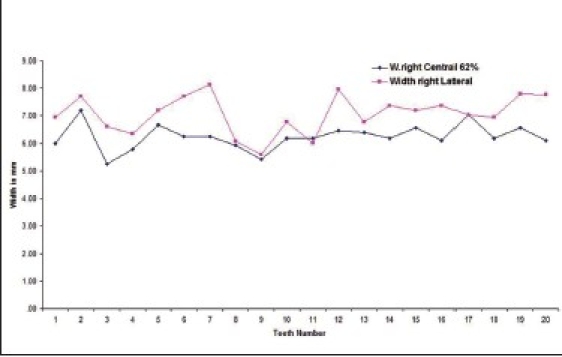
Golden proportion relation between right central incisor (blue line) and right lateral incisor (red line) in male subjects.

**Graph 6:**
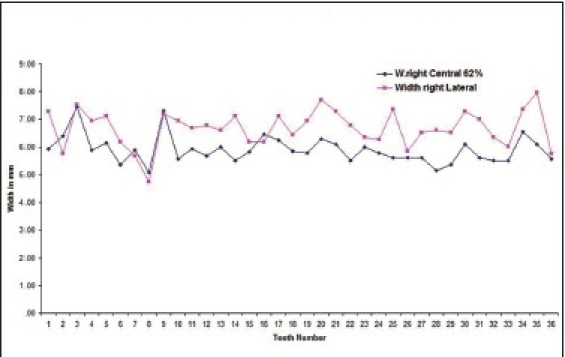
Golden proportion relation between right central incisor (blue line) and right lateral incisor (red line) in female subjects subjects.

The percentage that showed right central incisor in golden proportion to right lateral incisor [Graphs 5 and 6 respectively] was 16.1 (out of the total subjects).

**Graph 7:**
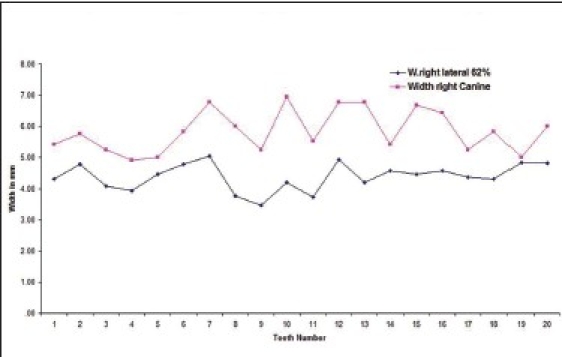
Golden proportion relation between right lateral incisor (blue line) and right canine (red line) in male subjects

**Graph 8:**
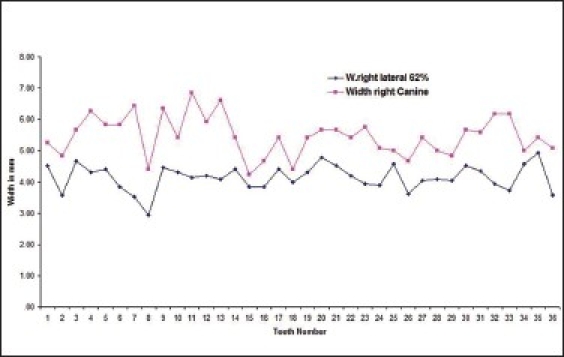
Golden proportion relation between right lateral incisor (blue line) and right canine (red line) in female subjects

The number of subjects with right lateral incisor in golden proportion right canine [Graphs 7 and 8 respectively] was 14.3% of the total.

The mean values and standard deviation for RED proportions for males and females are listed in Table 2. RED proportion between central incisor and lateral incisor lie in the 69.50-70.33% range. RED proportion between canine and lateral incisor lie in the 80-83% range.

**Table 2:** Indicates Red proportion relation between central incisor, lateral incisor and canine

		N	Mean	Std deviation	Std error	Minimum	Maximum
Red proportion lateral incisor/Central incisor left side	Male	20	69.91	8.28	1.85	55.90	84.08
	Female	36	69.50	6.94	1.16	45.71	80.81
	Total	56	69.65	7.38	.99	45.71	84.08

Red proportion cannine/Lateral insisor left side	Male	20	82.21	9.08	2.03	68.18	98.45
	Female	36	79.98	9.59	1.60	58.10	103.83
	Total	56	80.77	9.39	1.25	58.10	103.83

Red proportion lateral Incisor/central insisor right side	Male	20	70.33	6.04	1.35	60.16	80.65
	Female	36	70.44	6.45	1.13	55.76	81.35
	Total	56	70.40	6.75	.86	55.76	81.35

Red proportion Cannine/Lateral Insisor Right Side	Male	20	83.25	10.61	2.39	64.18	102.51
	Female	36	82.48	11.20	1.87	67.84	113.40
	Total	56	82.76	10.92	1.46	64.18	113.40

The values obtained for golden percentage, beginning with the right side canine and moving to the left canine, in this study were 12.5, 15.5, 22, 22, 15.5 and 12.5%.

**Graph 9:**
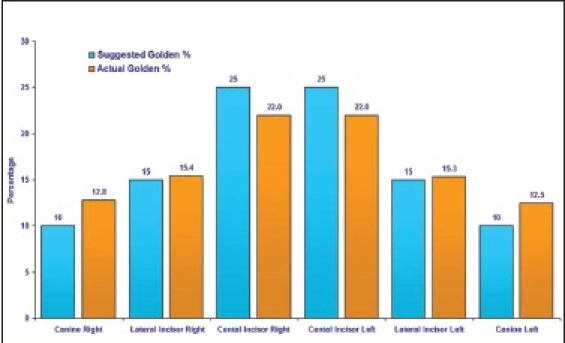
The relationship between the suggested golden percentage by Snow and values acquired in this study in male subjects

**Graph 10:**
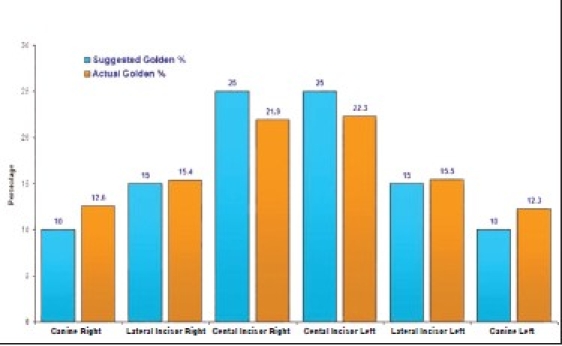
The relationship between the suggested golden percentage by Snow and values acquired in this study in female subjects

Graphs 9 and 10 show the relationship between the golden percentage suggested by Snow and the actual percentage for each anterior tooth for men and women respectively.

## DISCUSSION

It is important to determine a mathematical or geometrical relationship between teeth, in order to achieve an esthetic restorative result. It would be helpful if statistically reliable relationships existed to support the existing relationship theories.

This study was conducted on 56 dental students, 20 being male subjects and 36 female subjects. With respect to the theory of golden proportion, the best results in this study were seen in relation to perceived left lateral incisor width and perceived left canine width as seen from front. This was observed in a total of 14 (25%) out of 56 subjects, of which three (15%) were male subjects and 11 (30.6%) were female subjects.

The overall results showed that the golden proportion did not seem to exist. This was in accordance with the studies conducted by Minoo Mahshid *et al* and Fayyad MA *et al.* In their study of subjects with esthetic smile, they evaluated the existence of golden proportion by measuring the mesio-distal width of six anterior teeth, on scanned pictures of individuals. They arrived at the conclusion that golden proportion did not exist in natural dentition.[6,7]

With respect to RED proportion, the results of this investigation showed that the ratio of the width of maxillary lateral incisors to the width of central incisors is between 69.5 and 70.3%. The ratio of width of canine to width of lateral incisor is between 80 and 83%. In the present study, the ratio between central and lateral incisors and between lateral incisor and canine is not constant. The ratio increases as one moves distally.

The value 69.5-70.3%, which was the ratio of the width of maxillary lateral incisors to width of central incisors, is in agreement with the 70% RED proportion suggested by Ward,[4] and the mean proportion suggested by Fayyad *et al*,[7] which was between 66 and 78%.

The ratio between central and lateral incisors and between lateral incisor and canine is not constant, as suggested by Ward[4]

Hence, there is no evidence in this study to support the RED proportion theory as applied to natural dentition.

As for using Golden percentage theory to correlate the six anterior teeth, the result of the present investigation suggests that the mean values for golden percentage for central incisor is 21.9-22.3%. The mean value for lateral incisors is 15.3-15.5%. With respect to golden percentage of canines, the result of this study showed a mean value of 12.0-12.6%.

The values for lateral incisor was in agreement with those suggested by Snow,[5] who recommended a value of 15 as the golden percentage for lateral incisor.

The figures obtained for central incisor are slightly lower than those suggested by Snow,[5] who estimated 25% for central incisors.

Canines have a slightly higher value than those suggested by Snow,[5] who recommended a golden percentage value of 10 for canines.

In general, it appears that the width of central incisors is slightly smaller and the width of canines is slightly larger than those suggested by the golden percentage theory. A value of 22% for centrals, 15.5% for laterals, and 12.5% for canines can be adopted, as these percentages are more applicable to the natural dentition. Minor variations in the values obtained in this study, as compared to previous studies,[5] may be attributed to the ethnic difference of the subjects that were chosen in the present study.

## CONCLUSION

In the light of the results of this investigation the following conclusions can be derived:

1. The theory of Golden percentage was more applicable to the subjects of this study.
2. The golden proportion was not found to exist between perceived maxillary anterior teeth on natural dentition.
3. RED proportion was not found to exist between the six maxillary anterior teeth.
4. In order to establish objectively quantifiable width ratio between maxillary anterior teeth, ethnic differences should be taken into consideration. This will also help determine exactly what percentages are truly golden.
